# A comparison of static and dynamic ∆*B*_0_ mapping methods for correction of CEST MRI in the presence of temporal *B*_0_ field variations

**DOI:** 10.1002/mrm.27750

**Published:** 2019-03-28

**Authors:** Esau Poblador Rodriguez, Philipp Moser, Barbara Dymerska, Simon Robinson, Benjamin Schmitt, Andre van der Kouwe, Stephan Gruber, Siegfried Trattnig, Wolfgang Bogner

**Affiliations:** ^1^ High Field MR Center, Department of Biomedical Imaging and Image‐Guided Therapy Medical University Vienna Vienna Austria; ^2^ Medical Physics and Bioengineering University College London London United Kingdom; ^3^ Siemens Healthineers Sydney Australia; ^4^ Athinoula A. Martinos Center for Biomedical Imaging, Department of Radiology Massachusetts General Hospital, Harvard Medical School Boston Massachusetts; ^5^ Christian Doppler Laboratory for Clinical Molecular MR Imaging Vienna Austria

**Keywords:** ∆*B*_0_ mapping, chemical exchange saturation transfer (CEST), dynamic ∆*B*_0_ correction, frequency drift, scanner instabilities

## Abstract

**Purpose:**

To assess the performance, in the presence of scanner instabilities, of three dynamic correction methods which integrate ∆*B*_0_ mapping into the chemical exchange saturation transfer (CEST) measurement and three established static ∆*B*_0_‐correction approaches.

**Methods:**

A homogeneous phantom and five healthy volunteers were scanned with a CEST sequence at 7 T. The in vivo measurements were performed twice: first with unaltered system frequency and again applying frequency shifts during the CEST acquisition. In all cases, retrospective voxel‐wise ∆*B*_0_‐correction was performed using one intrinsic and two extrinsic [prescans with dual‐echo gradient‐echo and water saturation shift referencing (WASSR)] static approaches. These were compared with two intrinsic [using phase data directly generated by single‐echo or double‐echo GRE (gradient‐echo) CEST readout (CEST‐GRE‐2TE)] and one extrinsic [phase from interleaved dual‐echo EPI (echo planar imaging) navigator (NAV‐EPI‐2TE)] dynamic ∆*B*_0_‐correction approaches [allowing correction of each Z‐spectral point before magnetization transfer ratio asymmetry (*MTR_asym_*_)_ analysis].

**Results:**

All three dynamic methods successfully mapped the induced drift. The intrinsic approaches were affected by the CEST labeling near water (∆*ω* < |0.3| ppm). The *MTR_asym_* contrast was distorted by the frequency drift in the brain by up to 0.21%/Hz when static ∆*B*_0_‐corrections were applied, whereas the dynamic ∆*B*_0_ corrections reduced this to <0.01%/Hz without the need of external scans. The CEST‐GRE‐2TE and NAV‐EPI‐2TE resulted in highly consistent *MTR_asym_* values with/without drift for all subjects.

**Conclusion:**

Reliable correction of scanner instabilities is essential to establish clinical CEST MRI. The three dynamic approaches presented improved the ∆*B*_0_‐correction performance significantly in the presence of frequency drift compared to established static methods. Among them, the self‐corrected CEST‐GRE‐2TE was the most accurate and straightforward to implement.

## 1 INTRODUCTION

High and ultrahigh static magnetic fields (*B*_0_) provide advantages that are critical for the quality of CEST MRI results. Not only the SNR, but also the chemical specificity are significantly improved, because the spectral separation between the resonances of interest increases. The CEST also benefits from a prolonged storage of saturation in bulk water due to longer T_1_, which facilitates its sensitivity. Furthermore, CEST works particularly well in the slow to intermediate exchange regime (*k*_sw_ < ∆*ω*_S_, where *k*_sw_ is the exchange rate from the labile proton pool to the bulk water pool and ∆*ω*_S_ the solute proton pool frequency offset (which is proportional to *B*_0_)). Under this condition, spectral resonances of interest can be much better distinguished from the direct saturation of water. This regime is increasingly met at higher *B*_0_ even for rapidly exchanging protons.^1^, ^2^

On the other hand, local *B*_0 _inhomogeneities (∆*B*_0_) are more severe at higher *B*_0_, causing regionally dependent frequency shifts in Z‐spectra. This complicates quantification in CEST. The applied frequency‐selective CEST saturation pulses at ∆*ω*_RF_ are shifted away from the targeted nominal frequency offset (∆*ω*) by *δω* = ∆*ω*_RF _‐ ∆*ω* = ∆*B*_0_/γ (γ being the gyromagnetic ratio), which is proportional to the local ∆*B*_0_.^2^ Even with optimized *B*_0_ shimming protocols prior to the experiments, a retrospective ∆*B*_0_ correction is generally needed, in which the Z‐spectrum of each voxel is centered at the water resonance (0 ppm). A highly accurate ∆*B*_0_ correction is particularly important when studying exchanging compounds very close to water (e.g., glucose, lactate, myo‐inositol).^3^, ^4^, ^5^, ^6^ Zaiss et al. recently reported substantial pseudo‐CEST effects for dynamic CEST at 3 T from *B*_0_ alterations (~1% per 7‐Hz drift).^7^

The simplest approach for a ∆*B*_0_ correction is to determine the water resonance frequency from the Z‐spectrum (from here on this approach is termed “CEST‐minZ”).^8^, ^9^, ^10^ This works well only when the applied saturation power is low and the magnetization transfer contrast from semisolid macromolecules and CEST effect close to the water resonance can be neglected.^8^, ^9^, ^11^ In addition to this limitation, CEST‐minZ requires the full sampling of a high‐spectral‐resolution Z‐spectrum, which increases the scan time (i.e., the spectral range covering the water peak cannot be excluded). In vivo CEST measurements with higher saturation power and more accurate ∆*B*_0_ correction can be achieved by acquiring an external ∆*B*_0_ map. Water saturation shift referencing is probably the most widely used method to acquire such an external ∆*B*_0_ map for CEST data processing. In WASSR, a matching CEST pulse sequence is acquired as a prescan, but with low saturation power and targeting only a narrow range of offsets around 0 ppm with high spectral resolution.^12^ Alternatively, a prescanned ∆*B*_0_ map can be obtained from the phase difference of at least two gradient‐echo images acquired at different echo times (here termed “GRE‐2TE”).^13^, ^14^, ^15^, ^16^, ^17^, ^18^

All these three established ∆B_0_ correction methods, (A) CEST‐minZ, (B) WASSR, and (C) GRE‐2TE, estimate the water resonance frequency for a single time point and correct every z‐spectral point by applying the same shift (*δω*). This shift may not necessarily be representative for all points of the CEST spectrum, as they are acquired at different times. Consequently, all three methods share the drawback of being prone to errors due to temporal changes in the *B*_0_ field over the course of a CEST experiment. Such temporal ∆*B*_0_ fluctuations may arise from system instabilities [primarily heating of magnet’s gradient coils by heavy duty cycles^19^ or heating of passive shims^20^], from cardiac or respiratory effects^21^ or subject movement.^22^ Some previous studies performed at 3 T have reported drifts ranging from of 1.2 Hz/min to 5 Hz/min after a series of functional MRI or diffusion weighted imaging scans.^23^, ^24^, ^25^ Larger drifts are expected at higher *B*_0_ (e.g., at 7 T periodic *B*_0_ fluctuations of up to ~4 Hz due to respiration are already detectable even far away from the lungs).^26^, ^27^, ^28^, ^29^

In this paper, three dynamic ∆*B*_0_ correction methods, which integrate the ∆*B*_0_ mapping as part of the CEST sequence, are proposed; two of them via the phase generated by the CEST readout itself and the third by an interleaved 2D EPI navigator. They allow temporal fluctuations in *B*_0_ to be mapped and compensated for each individual Z‐spectral point separately. The accuracy of this *B*_0_ mapping and its impact on CEST correction are compared for these dynamic approaches and to three established static methods (i.e., CEST‐minZ, WASSR, and GRE‐2TE) for CEST analysis close to water.

## 2 THEORY

### 2.1 Chemical exchange in the presence of *B*_0_ inhomogeneities

At present, the most common CEST quantification metric is the asymmetric magnetization transfer ratio (*MTR_asym_*). The purpose of CEST asymmetry analysis is to separate the asymmetric CEST contribution from the symmetric components (e.g., direct water saturation).^30^, ^31^, ^32^ This analysis is performed by subtraction of the magnitude signal on one side of the Z‐spectrum from its mirrored side^1^, ^2^:

(1) $$MTR_{\mathrm{asym}}\left(\Delta \omega \right)=MTR\left(\Delta \omega \right)-MTR\left(-\Delta \omega \right)=\frac{S_{\mathrm{sat}}\left(-\Delta \omega \right)-S_{\mathrm{sat}}\left(+\Delta \omega \right)}{S_{0}},$$

where *S*_0_ and *S*_sat_ correspond to reference and labeled magnitude signals, respectively. Here the *MTR_asym_* would be equal to the proton transfer ratio expression considered in the two‐pool exchange model if the water saturation were caused purely by exchange and in the absence of ∆*B*_0_.^33^, ^34^, ^35^ However, when the frequency is shifted by *δω*, the proton transfer ratio can be redefined as^36^:

(2) $$PTR\left(\Delta \omega \right)=\eta \;\cdot \;PTR^{\prime }\left(\Delta \omega \right)=\eta \;\cdot \;\left(PTR_{\text{asym}}\left(\Delta \omega \right)-\Delta MTR\right),$$

where η is a modulation factor that fully compensates the proton transfer ratio and ∆MTR is the MTR offset used to compensate *MTR_asym_*. The factor η can be further derived as:

(3) $$\eta =\frac{\alpha \left(B_{1},\;\;\Delta \omega_{S}\right)\;\cdot \;\left(1-\sigma \left(B_{1},\;\;\Delta \omega_{S}\right)\right)}{\alpha \left(B_{1},\;\;\Delta \omega_{S}+\delta \omega \right)\;\cdot \;\left(1-\sigma \left(B_{1},\;\;\Delta \omega_{S}+\delta \omega \right)\right)}$$

It can be seen from Equation (3) that the modulation comes from the labeling coefficient *α* and spillover factor *σ*. In the denominator, both terms are dependent on the frequency shift *δω* with respect to the solute resonance frequency offset ∆*ω*_S_, making η strongly sensitive to Δ*B*_0_. It has previously been shown that inaccurate ∆*B*_0_ corrections can be erroneously interpreted as CEST effects, especially at frequencies close to the water resonance where the slope of the Z‐spectrum is steep because of direct saturation of bulk water.^37^

### 2.2 Static ∆*B*_0_ correction

The three state‐of‐the‐art static approaches for ∆*B*_0_ correction compared in this study are:

(A) CEST‐minZ

The water resonance frequency is intrinsically determined from the Z‐spectrum of each voxel (i.e., using magnitude images from a CEST experiment), by finding the minimum signal intensity value after a smoothing‐splines interpolation in the spectral domain.^9^, ^10^, ^38^

(B) WASSR

The WASSR uses an additional CEST sequence (i.e. prescan) with high spectral resolution over a narrow frequency range and low saturation power, in which CEST and MTC contributions are considered negligible, allowing the reference frequency to be estimated from the dominating direct water saturation. Similar to CEST‐minZ, WASSR utilizes only magnitude images, but the water resonance is determined by fitting the water peak with a Lorentzian curve, from which the central frequency of water can be estimated.^12^

(C) GRE‐2TE

**Figure 1:**
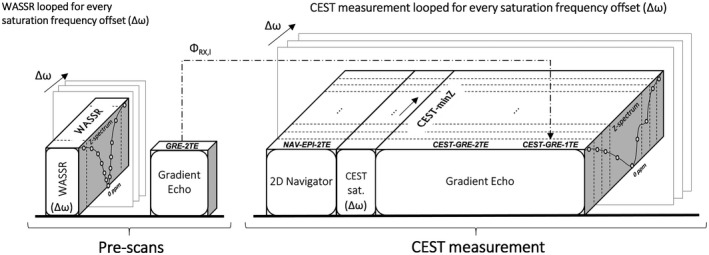
An overview of the sequence scheme for prescans and CEST measurement. The name of each ∆*B*_0_ mapping method appears on the top face of each block. The blocks represent image acquisition or labeling modules. The static correction methods WASSR and GRE‐2TE use only prescans (labeled correspondingly) to calculate ∆*B*_0_ maps. The CEST measurement is composed of a multishot 2D EPI navigator scan with dual‐echo readout; a CEST preparation in which the saturation RF pulses are applied at a range of frequency offsets depicted in the frequency axis of the Z‐spectra; and a gradient‐echo with dual‐echo readout. Magnitude and phase images from the interleaved navigator are used by the dynamic method NAV‐EPI‐2TE to generate a set of ∆*B*_0_ maps per ∆*ω*. In addition to their use for CEST quantification, CEST‐weighted images generated by the postlabeling gradient‐echo readout are used for self‐correction by three correction methods: 1) calculating one ∆*B*_0_ map using the static method CEST‐minZ (from the averaged magnitude images at TE_1_ and TE_2_); 2) computing a ∆*B*_0_ map for each ∆*ω* by the dynamic method CEST‐GRE‐2TE (from magnitude and phase images at echo times TE_1_ and TE_2_), and 3) calculating a ∆*B*_0_ map for each ∆*ω* by the dynamic method CEST‐GRE‐1TE (from magnitude and phase images at only the first echo time TE_1_). Note that CEST‐GRE‐1TE requires phase offset maps per channel (Φ_RX,I_) from the GRE prescan (indicated by dotted arrow). CEST, chemical exchange saturation transfer; CEST‐GRE‐2TE, chemical exchange saturation transfer‐gradient echo readout‐2TE; NAV, navigator

The GRE‐2TE approach calculates the ∆*B*_0_ maps from the difference of two gradient‐echo‐based phase images acquired at echo times TE_1_ and TE_2_ 13:

(4) $$\Delta B_{0}=\frac{\Delta \Phi }{\gamma \cdot \Delta TE}=\frac{\Phi_{TE_{2}}^{\text{GRE}}-\Phi_{TE_{1}}^{\text{GRE}}}{\gamma \;\cdot \;\left(TE_{2}-TE_{1}\right)},$$

where $\Phi_{TE_{j},l}^{\text{GRE}}$ is the phase image acquired at echo time (*j)*, by channel (*l*), in this case from the gradient‐echo pre‐scan (see Figure 1). For multichannel coils, the coil combination can be performed calculating the sum over the channels of the weighted channel‐wise phase difference (i.e., the sum Hermitian‐inner product)^14^:

(5) $$\Delta B_{0}=\frac{∠\sum_{l=1}^{\mathrm{Channels}}M_{TE_{2},l}^{\text{GRE}}\;\cdot \;M_{TE_{1},l}^{\text{GRE}}\;\cdot \;e^{i\left(\Phi_{TE_{2},l}^{\text{GRE}}-\Phi_{TE_{1},l}^{\text{GRE}}\right)}}{\gamma \;\cdot \;\left(TE_{2}-TE_{1}\right)},$$

where ∠ symbolizes the angle of the complex data, $M_{TE_{1},l}^{\mathrm{GRE}}$ and $M_{TE_{2},l}^{\mathrm{GRE}}$ are the magnitude images, and $\Phi_{TE_{1},l}^{\text{GRE}}$ and $\Phi_{TE_{2},l}^{\text{GRE}}$ are the phase images for a given channel acquired at echo times TE_1_ and TE_2_.

### 2.3 Dynamic ∆*B*_0_ correction

The three methods that we propose to use for CEST ∆*B*_0_ correction integrate dynamic B_0_ estimation as part of the CEST sequence, construct a ∆*B*_0_ map for each saturation frequency offset (∆*ω*) applied in the CEST labeling module, and allow the independent ∆*B*_0_ correction of each individual Z‐spectral point.

(D) CEST‐GRE‐2TE

The CEST‐GRE‐2TE method calculates the ∆*B*_0_ maps from multichannel dual‐echo data in the same way as the static method GRE‐2TE. The main difference is that the commonly used single‐echo readout of the CEST sequence is replaced by a dual‐echo readout. Thereby, the magnitude/phase from the prescan in Equation (5) is replaced by the CEST data, which are intrinsically generated immediately after the CEST‐labeling module (see Figure 1).

(E) CEST‐GRE‐1TE

Similar to CEST‐GRE‐2TE, this method derives ∆*B*_0 _maps from phase data from the CEST sequence, although in this case only a single echo is needed. This is possible if we assume that the phase offset per channel (Φ_RX,l_) is time‐invariant, in which case a single estimation of Φ_RX,l_, calculated from a dual‐echo prescan (e.g., the same used for method GRE‐2TE) is sufficient. This has been demonstrated to hold even at 7 T and where there is significant head motion.^39^, ^40^ The phase offsets are calculated using:

(6) $$\Phi_{RX,l}=\frac{TE_{1}\;\cdot \;\Phi_{TE_{2},l}^{\text{GRE}}-TE_{2}\;\cdot \;\Phi_{TE_{1},l}^{\text{GRE}}}{TE_{1}-TE_{2}}$$

This approach enables the calculation of dynamic ∆*B*_0_ maps from single‐echo readouts by correcting the phase images per channel before calculating the weighted averaged phases over the channels. The ∆*B*_0_ maps can hence be derived as:

(7) $$\Delta B_{0}\left(\Delta \omega \right)=\frac{∠\sum_{l=1}^{\mathrm{Channels}}M_{TE_{1,l}}^{\text{CEST}-\text{GRE}}\left(\Delta \omega \right)\;\cdot \;e^{i\left(\Phi_{TE_{1,l}}^{\text{CEST}-\text{GRE}}\left(\Delta \omega \right)-\Phi_{RX,l}\right)}}{\gamma \;\cdot \;TE_{1}},$$

where $M_{TE_{1,l}}^{\text{CEST}-\text{GRE}}\left(\Delta \omega \right)$ and $\Phi_{TE_{1,l}}^{\text{CEST}-\text{GRE}}\left(\Delta \omega \right)$ are the magnitude and phase images per channel, acquired from the CEST readout at a single echo time for each frequency offset.

(F) NAV‐EPI‐2TE

In contrast to using the intrinsic magnitude/phase information of the CEST‐weighted images, the NAV‐EPI‐2TE method uses dual‐echo data in Equation (5), which are additionally acquired before each saturation module via a 2D multishot EPI navigator (see Figure 1).

## 3 METHODS

The accuracy of all six ∆*B*_0_ correction methods and any possible bias on the intrinsic dynamic mapping methods (i.e., CEST‐GRE‐2TE and CEST‐GRE‐1TE) was first investigated in a homogeneous polydimethylsiloxane oil phantom (Siemens AG, Munich, Germany). In such a phantom, *MTR_asym_* should ideally be 0% in the absence of magnetization transfer (MT) from semisolid or CEST agents. Subsequently, each method was tested on five healthy volunteers (three males, two females; mean age 34 ± 4 years) after Ethics Committee approval by the Medical University of Vienna and informed consent was obtained.

All phantom scans were performed for a single slice with 1.7 × 1.7 × 6 mm^3^ spatial resolution over a field of view of 220 × 220 mm^2^ with a spectral resolution of 0.11 ppm. For the volunteer scans, the field of view was 270 × 270 mm^2^ with a resolution of 2.1 × 2.1 × 6 mm^3^, and the frequency offset increments were 0.15 ppm. Details on imaging parameters are listed in the following and in Table 1. Imaging parameters were matched wherever possible.

**Table 1:** Comparison of main scan parameters for each of the ∆*B*_0_ acquisition methods

	∆*B*_0_ mapping	TR [ms]	TE1 [ms]	TE2 [ms]	BW [Hz/Px]	k‐space lines / shot	Miscellaneous
**STATIC**	**CEST‐minZ**	9.5	1.74	‐	780	1	Ts = 700 ms, B_1rms _= 2.0 µT, ∆*ω *= 0.15/0.11 ppm
	**WASSR**	4.5	1. 74	‐	780	1	Ts = 100 ms, B_1rms _= 0.2 µT, ∆*ω *= 0.05/0.01 ppm
	**GRE‐2TE**	9.5	1.74	5.16	780	1	
**DYNAMIC**	**CEST‐GRE‐2TE**	9.5	1.74	5.16	780	1	Ts = 700 ms, B_1rms _= 2.0 µT, ∆*ω *= 0.15/0.11 ppm
	**CEST‐GRE‐1TE**	9.5	1.74	‐	780 (390)*	1	Ts = 700 ms, B_1rms _= 2.0 µT, ∆*ω *= 0.15/0.11 ppm
	**NAV‐EPI‐2TE**	15	5.4	9.0	2442	4	

* The experimentally used readout BW of 780Hz/Px stated here for CEST‐GRE‐1TE, could be in practice halved to 390Hz/Px, thereby matching the readout duration of two echoes of the CEST‐GRE‐2TE readout. Averaging these two echoes of the CEST‐GRE‐2TE readout with 780Hz/Px BW, should result in very similar SNR as the single CEST‐GRE‐1TE readout with 390Hz/Px BW.

### 3.1 Static ∆*B*_0_ corrections

- To minimize the sensitivity to temporal instabilities, the Z‐spectral points were sampled with alternating saturation frequency offsets, decreasing from the maximum frequency to those close to water.
- For WASSR, a high spectral resolution with ∆*ω* steps of 0.05 ppm (≈15 Hz) over a frequency range of ±0.8 ppm was chosen, resulting in a total acquisition time of <4 min for in vivo and ∆*ω* = 0.01 ppm (≈3.6 Hz) with a total acquisition time <6 min for phantom measurements.
- For GRE‐2TE, the prescan was acquired in <1 s. To prevent possible phase errors from mistiming of readout gradients and the acquisition, both echoes were sampled under gradients of the same polarity (a.k.a. “monopolar”).

### 3.2 Dynamic ∆*B*_0_ corrections

**Figure 2:**
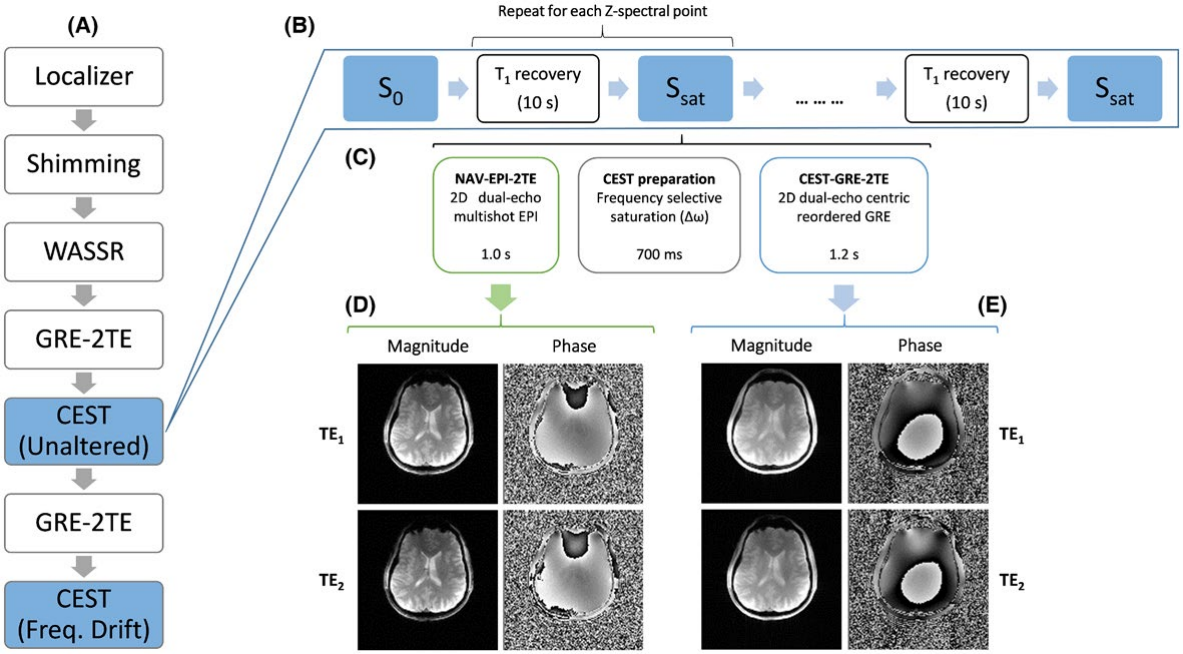
An overview of the experimental protocol followed for the comparison of six ∆*B*_0_ correction methods (A). Scheme of the CEST measurement (B), in which a delay for T1 recovery of water signal is interleaved with *S*_0_ and *S*_sat_ acquisitions, each of them consisting of three blocks (C): an EPI navigator with dual‐echo readout scan generating magnitude and phase images for each TE (D); a CEST labeling period in which the magnetization is saturated by a train of saturation pulses at a frequency offset ∆*ω* for each Z‐spectral point; and a GRE sequence with dual‐echo readout scan generating magnitude and phase images for each echo time (E). CEST, chemical exchange saturation transfer; EPI, echo planar imaging; GRE, gradient‐echo;WASSR, water saturation shift referencing

- For CEST‐GRE‐2TE, the more commonly used single‐echo readout of the CEST sequence was replaced by two readouts with doubled receiver bandwidth (e.g., 780 Hz/Px instead of 390 Hz/Px).

- The CEST‐GRE‐1TE could be acquired with no change to the conventional single‐echo readout of the CEST sequence. However, to prevent bias in the comparison, data from TE_2_ of the GRE readout post CEST labeling were simply ignored and the ∆*B*_0_ mapping used only data from TE_1_. The required coil offset maps, which were assumed to be time‐invariant,^39^, ^40^ were obtained from the GRE pre scan.

- For NAV‐EPI‐2TE, a navigator was placed before each CEST‐labeling module as shown in Figures 1 and 2. The number of k‐space lines (i.e., echoes) collected in each shot of the EPI navigator was set to 4 to minimize geometric distortions and a readout bandwidth of 2442 Hz/Px used to reduce fat‐water chemical shift to 0.4 mm, resulting in a ~1‐s navigator duration. This could be further shortened by increasing the number of k‐space lines per shot (e.g., from 4 up to 128). The delay between the ∆*B*_0_ mapping with the navigator and CEST data sampling illustrated in Figure 2 was assumed to be negligible.

### 3.3 Measurement protocols

All experiments were performed on a whole‐body 7 T MR Magnetom system (Siemens Healthcare, Erlangen, Germany) with a ^1^H 32‐channel head coil (Nova Medical, Wilmington, Massachusetts, USA). As illustrated in Figure 2A, after localization and *B*_0_ shimming, three prescans were performed: 1) One WASSR measurement, in which the magnetization preparation was performed using one saturation pulse (B_1rms_ = 0.2 µT) and 2) two monopolar dual‐echo gradient‐echo scans (duration <1 s), one prior to each CEST measurement, which generated magnitude and phase images for each channel and echo time.

To investigate the performance of the dynamic ∆*B*_0_ correction methods versus the static ones in the presence of scanner instabilities, a scan‐rescan experiment was defined. The first CEST acquisition was performed with no deliberate changes to the imaging system, while in the second one a linear frequency drift of 60 Hz [consistent with previously reported drifts up to 5 Hz/min at 3 T 23, 24, 25]) was induced over the duration of the scan. The sequence was modified to apply a drift by updating the reference frequency in the analog‐to‐digital converters (ADCs) blocks of the navigator and CEST readouts for each ∆*ω* loop.

Each CEST scan (of duration 13 min) comprised three blocks (Figure 2): 1) a multishot EPI navigator with dual‐echo readout “NAV‐EPI‐2TE”; followed by 2) the CEST‐labeling module; and 3) a train of monopolar dual‐echo gradient‐echo readouts that covered the entire k‐space in one step (i.e., “CEST‐GRE‐2TE”). A subsequent delay of 10 s ensured T_1_ recovery of the water signal between acquisitions of different Z‐spectral points. The CEST labeling was executed by a train of four Gaussian pulses of 100‐ms duration, duty cycle of 50%, and B_1rms_ = 2.0 µT. To study the bias of inaccurate Δ*B*_0_ correction (which should be particularly strong closer to water) 61 spectral offsets were equidistantly distributed in the range from −4.5 ppm to 4.5 ppm (−4.5 ppm, +4.5 ppm, −4.35 ppm, +4.35 ppm, …, 0 ppm) for in vivo and 41 spectral offsets from −2.2 ppm to 2.2 ppm for phantom measurements. Magnitude and phase images were saved separately for each channel and echo.

### 3.4 Data analysis

All MR images were saved in DICOM format and data processing and evaluation were conducted retrospectively with MATLAB (R2017b, MathWorks, Natick, MA USA).

The resonance frequency of bulk water was determined voxel‐wise for the static methods CEST‐minZ and WASSR, as the minimum of the spline‐interpolated z‐spectra,^9^ and by least squares Lorentzian fitting [MATLAB function b0wassr.m available at http://godzilla.kennedykrieger.org/CEST/], respectively. For the ∆*B*_0_ mapping methods (C)–(F) phase images from different coils were combined by applying Eqs. (5), (6), (7) and subsequently unwrapped via fast 2D phase unwrapping^41^ available at https://github.com/mfkasim91/unwrap_phase/. The phase offset maps required for the method CEST‐GRE‐1TE were masked and smoothed using a discretized spline smoother [MATLAB function smoothn.m^42^] to provide reliable results even at the brain’s boundaries, as has been shown previously for coil combination and distortion correction.^40^, ^43^, ^44^, ^45^ Finally, as the ∆*B*_0_ maps were not masked, they were smoothed by a spatial Hamming filter before being used for CEST correction.

Each pair of magnitude images (i.e., from the CEST double‐echo readout) were averaged and ∆*B*_0_ correction and subsequent *MTR_asym_* analysis [Equation (1)] were performed voxel‐wise. For static ∆*B*_0_ correction methods (A)–(C), the estimated frequency shift *δω* was applied to center the entire Z‐spectra to 0 ppm, whereas for the dynamic methods (D)–(F) a time‐dependent *δω*(*t*) was applied to correct each Z‐spectral point independently.

To evaluate the *B*_0_ estimation performance between different methods, a region of interest (ROI) was defined to compute ROI‐averaged *B*_0_ and *MTR_asym_* curves (Figure 6). To evaluate the effect of ∆*B*_0_ on the *MTR_asym_* maps, a ROI was manually drawn along phantom and volunteer’s brain boundaries and *MTR_asym_* mean and standard deviation were derived from voxels contained within these ROIs.

## 4 RESULTS

**Figure 3:**
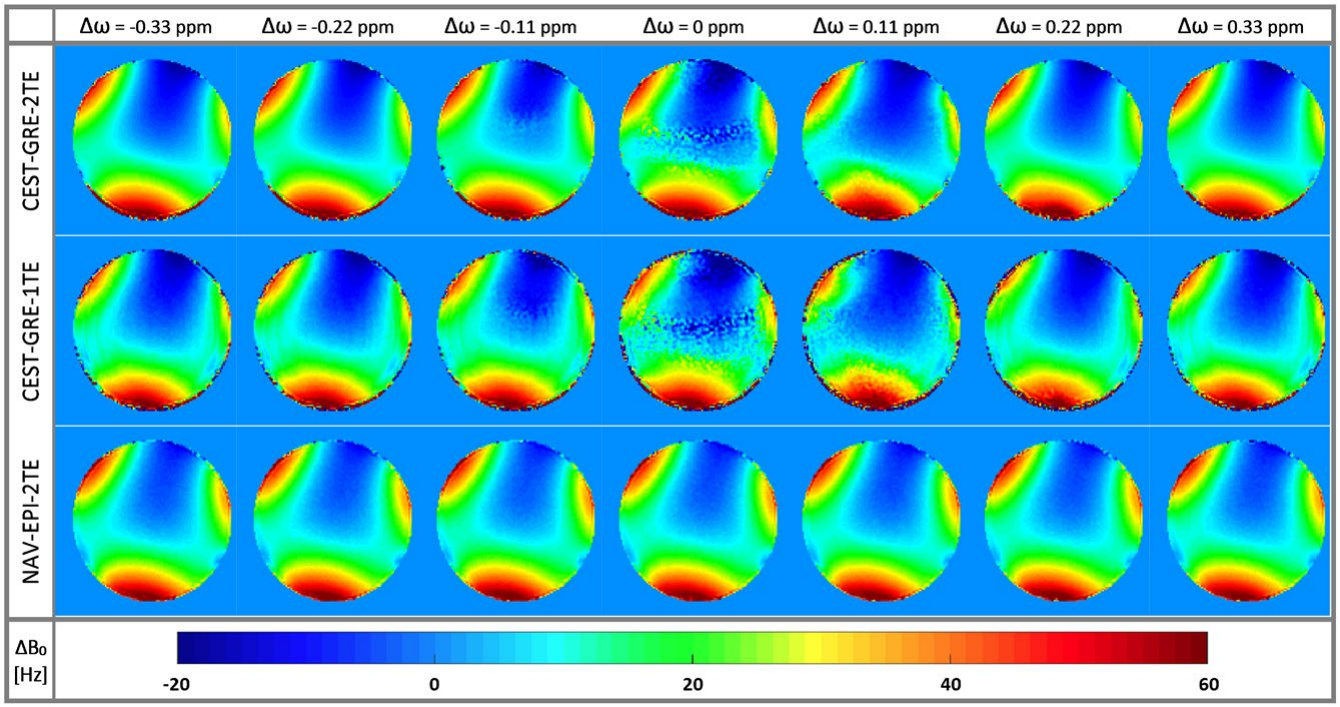
Color‐coded ∆*B*_0_ maps for the proposed dynamic methods CEST‐GRE‐2TE (first row), CEST‐GRE‐1TE (second row), and NAV‐EPI‐2TE (third row) acquired at seven saturation frequency offsets around the water resonance (0 ppm) at ∆*ω* = [‐0.33 to 0.33] ppm (columns). NAV‐EPI‐2TE is the only dynamic method insensitive to the RF saturation pulses applied for CEST labeling. Methods CEST‐GRE‐1TE and CEST‐GRE‐2TE are consistent with these results distant to the water resonance, but suffer from corrupted maps for ∆*ω* < |0.33| ppm (i.e., close to water). CEST‐GRE‐1TE, chemical exchange saturation transfer‐gradient‐echo‐1TE; EPI, echo planar imaging; GRE, gradient‐echo; NAV‐EPI‐2TE, navigator‐echoplanar imaging‐2TE

The accuracy of dynamic *B*_0_ estimation via CEST‐GRE‐2TE and CEST‐GRE‐1TE was compromised when the CEST‐labeling pulses were applied close to water. Figure 3 shows how the ∆*B*_0_ maps of phantom experiments were apparently corrupted by saturation RF trains applied at ∆*ω* < |0.33| ppm. On the other hand, NAV‐EPI‐2TE presented unaffected ∆*B*_0_ maps over the whole saturation ∆*ω* range.

**Figure 4:**
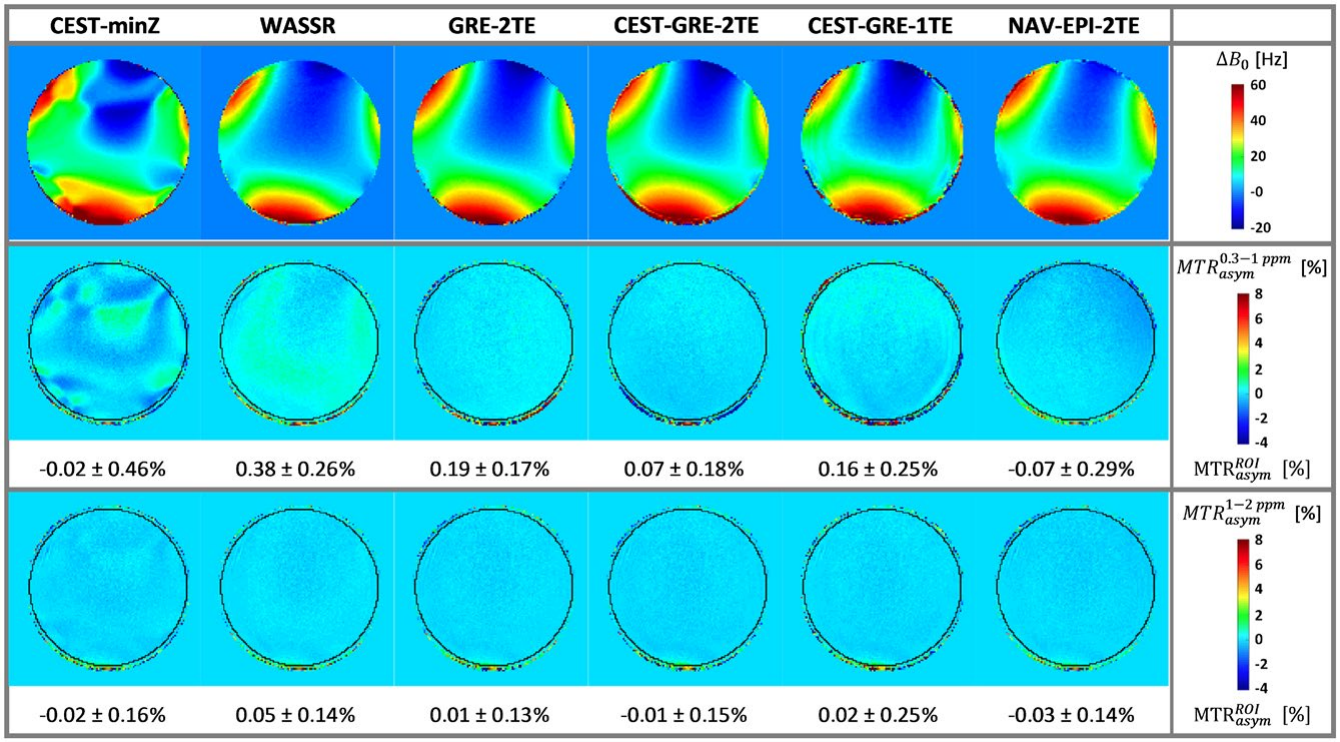
The ∆*B*_0_ mapping accuracy in terms of *MTR_asym_* in a homogeneous phantom: The first row presents the color‐coded ∆*B*_0_ maps determined by the static methods (CEST‐minZ, WASSR, and GRE‐2TE) and the first ∆*B*_0_ map from the dynamic methods (CEST‐GRE‐1TE, CEST‐GRE‐2TE, and NAV‐EPI‐2TE). Below color‐coded maps of ∆*ωB*_0_‐corrected *MTR_asym_* values are shown for the integration range ∆*ω* of ± [0.3–1.0] ppm (second row) and ±[1–2] ppm (third row). Values within the black circular delineated ROI are expressed as mean ± standard deviation. Accurate ∆*ωB*_0_ correction is indicated by homogeneous *MTR_asym_* maps with low values, as the phantom containing no MTC or CEST agents should result in *MTR_asym_* = 0%. Methods GRE‐2TE and CEST‐GRE‐2TE provided the most accurate *MTR_asym_* values among static and dynamic methods with similar low spatial variability. CEST, chemical exchange saturation transfer; EPI, echoplanar imaging; GRE, gradient‐echo; MTC, magnetization transfer contrast; *MTR_asym_*, asymmetric magnetization transfer ratio; NAV, navigator; ROI, region of interest; WASSR, water saturation shift referencing

Figure 4 presents the accuracy of ∆*B*_0_ maps for the different correction methods by evaluating deviations from the 0% *MTR_asym_* that was expected for a phantom containing no MTC or CEST agents. The GRE‐2TE generated the most homogeneous *MTR_asym_* map (i.e., although comparable to WASSR, the one that had the lowest spatial variability) among the static methods with minimal offset from 0% *MTR_asym_* compared to WASSR. Among the dynamic methods, CEST‐GRE‐2TE resulted in the most accurate *MTR_asym_* maps, with spatial variability similar to GRE‐2TE. The methods, CEST‐GRE‐1TE and NAV‐EPI‐2TE, on the other hand, led to slight spatial gradients in *MTR_asym_* maps, and ~40% to 60% higher variability than for CEST‐GRE‐2TE. The third row of Figure 2 illustrates that most of these differences become negligible for ∆*ω* = ±[1–2] ppm.

**Figure 5:**
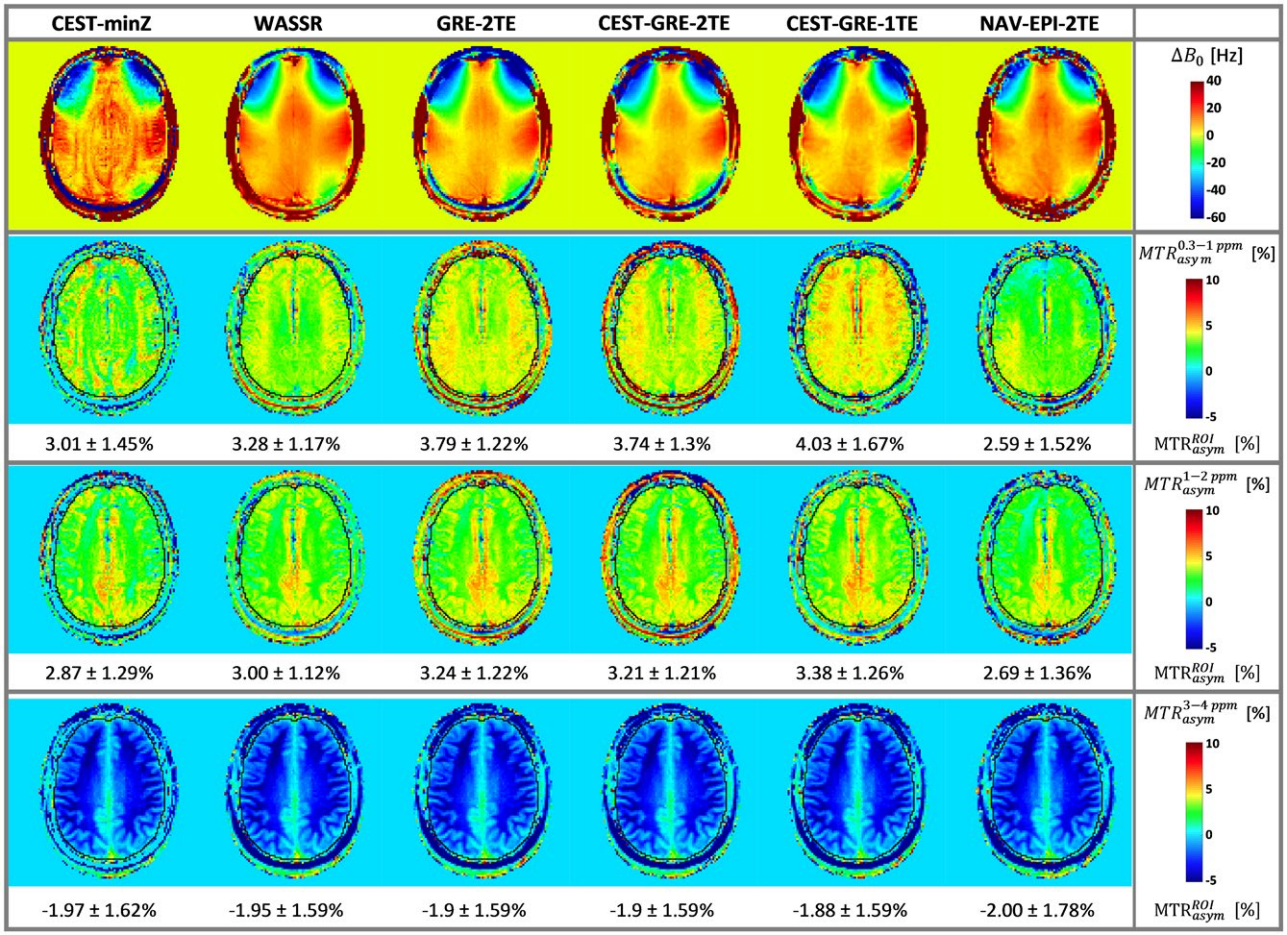
Performance of each ∆*B*_0_ correction method under unaltered conditions for volunteer V2: The first row shows the color‐coded ∆*B*_0_ maps of the static correction methods (CEST‐minZ, WASSR, and GRE‐2TE) and the first ∆*B*_0_ map from the dynamic methods (NAV‐EPI‐2TE, CEST‐GRE‐1TE, and CEST‐GRE‐2TE). Below, color‐coded maps of ∆*B*_0_‐corrected *MTR_asym_* values for the frequency ranges |0.3–1.0|, |1–2|, and |3–4| ppm are displayed in the second, third, and fourth rows, respectively. The outline of the brain, defining voxels included in the mean *MTR_asym_* (see Section “Data analysis”), is indicated in black. The dynamic method CEST‐GRE‐1TE appears to estimate slightly lower *B*_0 _compared to method CEST‐GRE‐2TE, producing a higher‐valued *MTR_asym_* map. The NAV‐EPI‐2TE shows the opposite effect (overestimation of *B*_0_ compared to CEST‐GRE‐2TE) in the frontal region. CEST‐minZ, determination of the water resonance frequency from the Z‐spectrum; GRE‐2TE, *MTR_asym_*, gradient echo‐2TE‐asymmetric magnetization transfer ratio; NAV‐EPI‐2TE,navigator‐echoplanar imaging‐2TE; WASSR, water saturation shift referencing

For in vivo experiments, MT effects and other confounding factors cannot be neglected, so ∆*B*_0_ mapping accuracy cannot be evaluated. However, rows 2 to 4 of Figure 5 show how the differences between correction methods gradually diminish the further the integration ranges ∆*ω* are from the water resonance. This indicates that in vivo CEST is sensitive to ∆*B*_0_ over a wider range of ∆*ω* than the phantom experiments. The ∆*B*_0_ corrections via static methods were generally inferior to those using the dynamic methods for ∆*ω* = ±[0.3–1.0] ppm (Figure 5). The CEST‐GRE‐1TE slightly underestimated *B*_0_ compared to CEST‐GRE‐2TE, thereby producing artificial *MTR_asym_* increases, while the *MTR_asym_* maps corrected by NAV‐EPI‐2TE showed a gradient from posterior left to anterior right direction (consistent with the phantom experiments).

**Figure 6:**
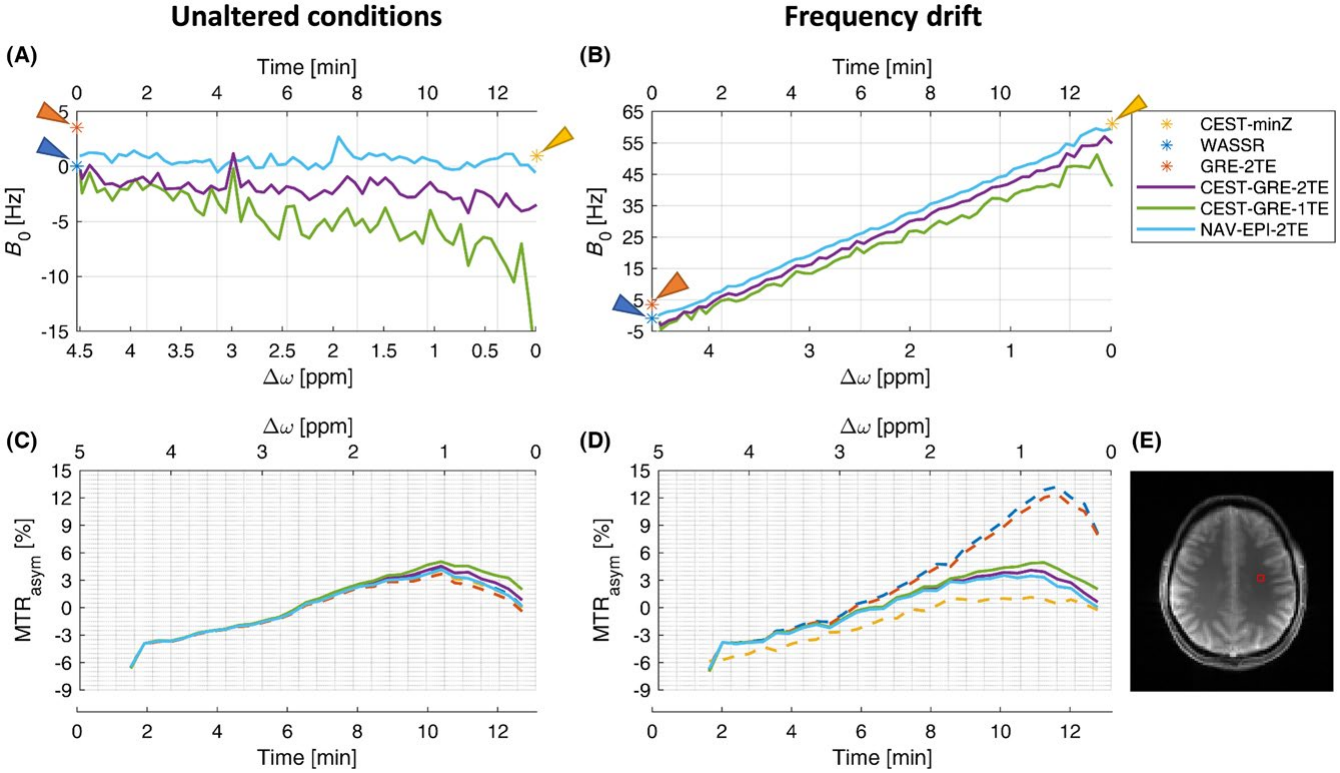
Dynamic field mapping and its impact on the *MTR_asym_* within an ROI located in the white matter (WM) section of volunteer V3 (E). The ROI‐averaged *B*_0_ estimation without (A) and with (B) an induced frequency drift of ~4.6 Hz/min. The static methods are represented by stars, while dynamic methods are depicted by solid lines. The times on the x axes are the times elapsed after the “GRE‐2TE” prescan. For illustration the time point of the WASSR acquisition was also set to *t* = 0. The offsets on the x axis ( ∆*ω*) refer to the frequencies at which saturation pulses were applied at the time instant when each *B*_0_ sample was determined. Differences in *MTR_asym_* curves with different ∆*B*_0_‐correction methods under unaltered (C) and drifted (D) conditions are shown in the bottom row. CEST‐GRE‐2TE (purple), CEST‐GRE‐1TE (green), and NAV‐EPI‐2TE (light blue) succeeded in mapping and correcting the induced drift ∆*B*_0_(*t*). A good estimate for the precision of the *B*_0 _mapping approaches can be derived from the standard deviation of the *B*_0_ measurements in absence of an induced frequency drift (A). For CEST‐GRE‐2TE, assuming a Gaussian distribution, it can be stated that in 95% of all cases a *B*_0_ change of ±1.92 Hz can be corrected. CEST‐GRE‐1TE, chemical exchange saturation transfer‐gradient echo‐1TE; *MTR_asym_*, asymmetric magnetization transfer ratio; NAV‐EPI‐2TE, navigator‐echoplaner imaging‐2TE; ROI, region of interest; WASSR, water saturation shift referencing

Figure 6 shows the variation in *B*_0_ over time and the effect of this on the corrected *MTR_asym_* curves within a ROI placed in a white matter (WM) region of volunteer V3, for both minimum field change and the imposed frequency drift. The dynamic mapping methods successfully followed the *B*_0_ evolution and corrected each Z‐spectral point independently before the *MTR_asym_* analysis. All static methods, on the other hand, resulted in severely underestimated (~1/3 for CEST‐minZ) or overestimated (~3 times for WASSR and GRE‐2TE) *MTR_asym_* values.

**Figure 7:**
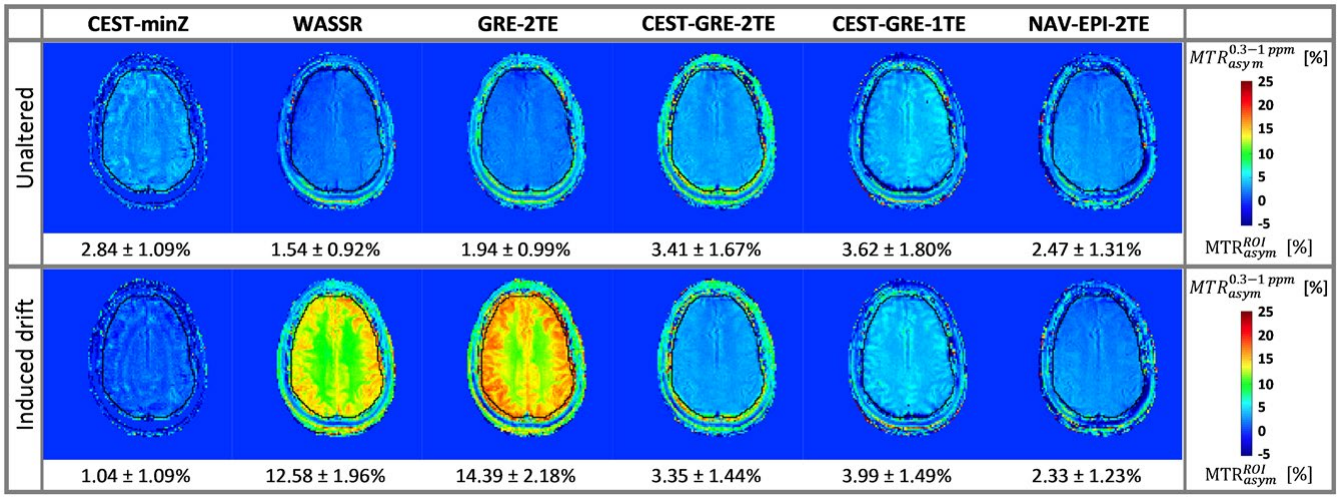
Effect of a known linear frequency drift on the *MTR_asym_* maps of volunteer V4: The top row shows color‐coded maps for the different correction methods in unaltered conditions (an apparent drift of ~6 Hz was measured between the GRE‐2TE prescan and the CEST acquisition at ∆*ω* = ±1 ppm); the bottom row depicts maps for which a ~4.6‐Hz/min drift was induced. The static method CEST‐minZ overestimated *B*_0_, resulting in decreased *MTR_asym_* values, while WASSR and GRE‐2TE show the opposite effect, since both scans were performed before the field drift was applied. Among the dynamic methods, CEST‐GRE‐2TE and NAV‐EPI‐2TE compensate for this drift most efficiently, providing very close *MTR_asym_* values within the delineated ROI (in black) between acquisitions, followed by CEST‐GRE‐1TE. CEST‐GRE‐2TE, chemical exchange saturation transfer‐gradient echo‐2TE; CEST‐minZ; determination of the water resonance frequency from the Z‐spectrum; *MTR_asym_*, asymmetric magnetization transfer ratio; NAV‐EPI‐2TE, navigator echoplanar imaging‐2TE; ROI, region of interest; WASSR, water saturation shift referencing

The Δ*B*_0_‐corrected color‐coded *MTR_asym_* maps derived from the scan‐rescan experiment are presented in Figure 7 for volunteer V4. For the CEST sequence with induced frequency drift, the *MTR_asym_*contrast after Δ*B*_0 _correction via CEST‐minZ was underestimated, while WASSR and GRE‐2TE Δ*B*_0_ correction led to the opposite effect. On the contrary, all dynamic methods compensated for this drift efficiently. The CEST‐GRE‐2TE and NAV‐EPI‐2TE achieved *MTR_asym_*maps with the most similar contrasts with and without induced frequency drift (ROI‐averaged errors of 0.001%/Hz and 0.002%/Hz drift, respectively), while CEST‐GRE‐1TE showed slightly higher deviation (e.g., error of 0.006%/Hz), but was still superior to all the static approaches.

**Figure 8:**
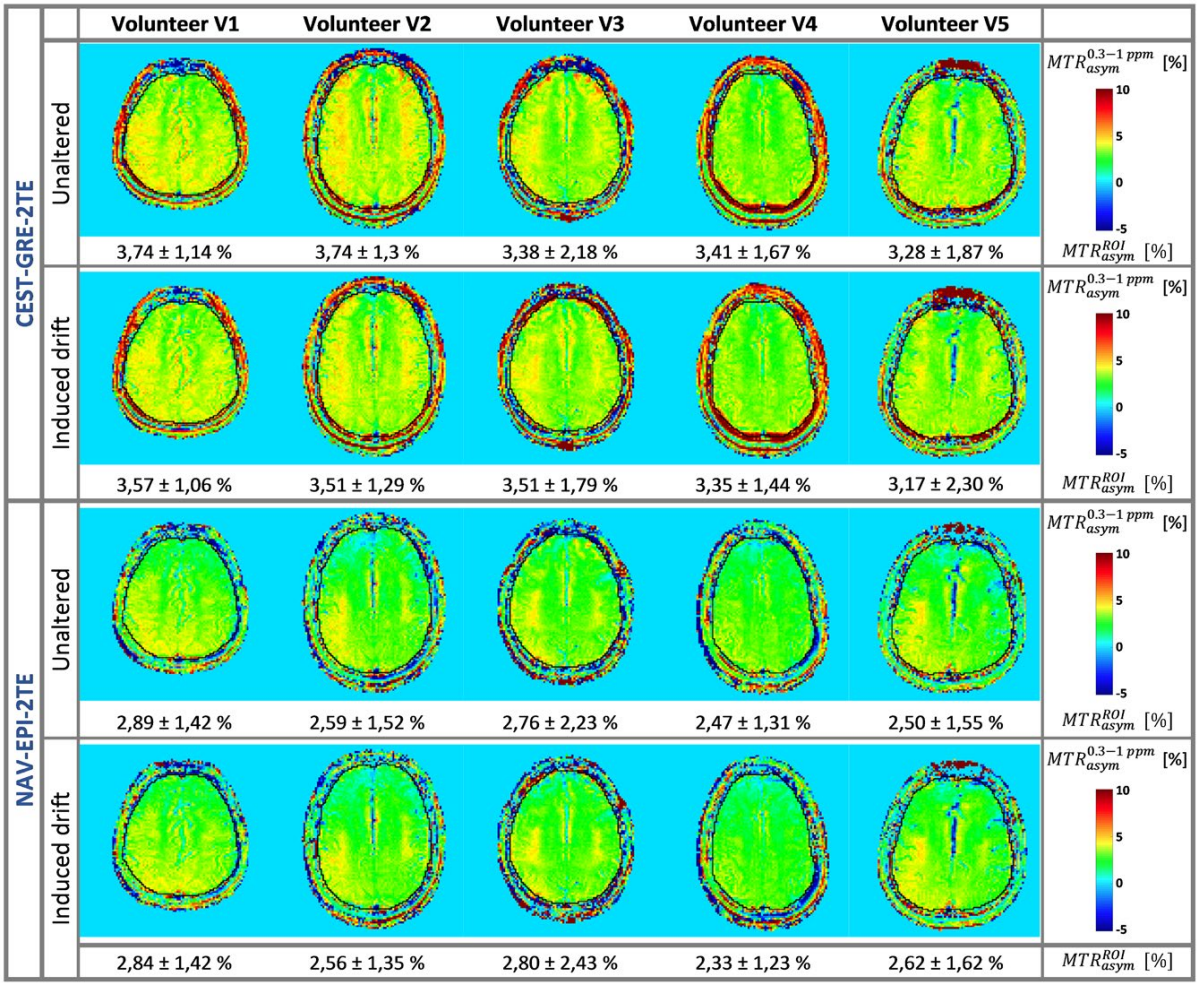
Comparison of the correction performance of the proposed dynamic methods CEST‐GRE‐2TE (rows 1 and 2) and NAV‐EPI‐2TE (rows 3 and 4) for subjects V1‐V5: ∆*B*_0_‐corrected *MTR_asym_* (0.3–1.0 ppm) maps in the absence (first and third rows) and in the presence (second and fourth rows) of the induced *B*_0_ drift during each CEST measurement. A ROI along each volunteer's brain border was manually drawn (delineated in black) and the *MTR_asym_* mean ± standard deviation values within this ROI are shown at the bottom of each map. Among the dynamic methods, both CEST‐GRE‐2TE and NAV‐EPI‐2TE generated highly consistent *MTR_asym_* contrasts between scans in all cases. CEST‐GRE‐2TE, chemical exchange saturation transfer‐gradient echo‐2TE; *MTR_asym_*, asymmetric magnetization transfer ratio; NAV‐EPI‐2TE, navigator‐echoplanar imaging‐2TE

The results of the comparison among all five healthy volunteers (V1‐V5) for the dynamic Δ*B*_0 _correction methods CEST‐GRE‐2TE and NAV‐EPI‐2TE are presented in Figure 8. The *MTR_asym_* maps derived by CEST‐GRE‐2TE and NAV‐EPI‐2TE were highly consistent between acquisitions with/without artificially induced frequency drift for all subjects.

## 5 DISCUSSION

In this study, we have investigated the applicability of three dynamic ∆*B*_0_ mapping methods for correction of CEST, which integrate ∆*B*_0_ mapping in the CEST measurement. In contrast to static correction methods, each Z‐spectral point can be adjusted independently to compensate for temporal *B*_0_ changes such as those arising from system instabilities. Static and dynamic correction performance were evaluated and compared focusing on the Δ*B*_0_ mapping accuracy and bias of *MTR_asym_* maps in the human brain at 7 T, first in the absence and then in the presence of a frequency drift.

Previous studies have evaluated the accuracy of static ∆*B*_0_ correction of CEST‐weighted maps by multiecho methods,^15^, ^16^, ^17^, ^18^ while studies proposing dynamic methods to correct for temporal ∆*B*_0_ changes have only recently emerged.^46^, ^47^

Windschuh et al. proposed a method to correct each Z‐spectral point independently retrospectively, combining phase images from a single‐echo GRE CEST readout and a prescan to calculate relative ∆*B*_0_ maps (WASABI).^46^ However, development work is still needed to improve the stability of this approach, which was significantly affected by the RF saturation pulses even at ∆*ω* ≈ 1 ppm from the water resonance. In contrast, the intrinsic dynamic correction methods we propose here (CEST‐GRE‐2TE and CEST‐GRE‐1TE) were affected by the CEST labeling only within a narrow range of ∆*ω* < |0.3| ppm, most likely because of low SNR of the saturated CEST images.

Windschuh et al. estimated the error of the *MTR_asym_* (1.1 ppm) to be on average 0.18%/Hz drift. This is in good agreement with the deviations of *MTR_asym_* (0.3–1.1 ppm) between acquisitions with and without induced *B*_0_ drift found in our study (e.g., 0.21%/Hz and 0.18%/Hz corrected by WASSR and GRE‐2TE, respectively). The spatial inhomogeneity cannot be directly compared, since the surface of the ROI over the measured cartilage on the knee in the study by Windschuh et al. was much smaller than that covered here, in the brain.

Simegn et al. proposed a prospective motion and ∆*B*_0_ correction of glycoCEST by updating the zero‐order and first‐order shim gradients for each CEST offset acquisition using a 3D version similar to that of our navigator.^47^ However, they did not show any ∆*B*_0_ map or provide any information about the remaining local inhomogeneities of higher than first‐order within the CEST volume after correction, and hence did not attempt to apply any further postprocessing steps.

The metric *MTR_asym_*, which is highly sensitive to frequency shift errors close to the water resonance,^12^ has been used to assess the quality of the ∆*B*_0_ correction methods in a similar way to the previously proposed Symmetric Analysis of Z‐Spectra (SAS).^48^ The static GRE‐2TE and the dynamic CEST‐GRE‐2TE methods resulted in *MTR_asym_* maps with lowest spatial variability and mean values closest to 0% for the phantom experiments. This indicates that these two methods lead to the most accurate ∆*B*_0_ correction among the static and dynamic approaches.

The in vivo results showed comparable corrected CEST‐weighted signal distribution between the static methods GRE‐2TE and WASSR. In contrast, the dynamic methods showed the following divergences: 1) the *MTR_asym_* maps corrected by GRE‐CEST‐2TE achieved similarly homogeneous distribution to the static methods; and 2) corrections performed by CEST‐GRE‐1TE and NAV‐EPI‐2TE resulted in an overall positive *MTR_asym_* offset and a slight spatial gradient in the anterior‐posterior direction (frequency encoding direction) relative to GRE‐CEST‐2TE, respectively. These effects could arise from delays of the applied gradients and could be corrected by acquiring the same scan with opposite image readout orientation.^49^, ^50^

The scan‐rescan experiment revealed very high consistency between Δ*B*_0_‐corrected *MTR_asym_* maps with and without frequency drift for all subjects when using GRE‐CEST‐2TE and NAV‐EPI‐2TE for all volunteers. The *B*_0_ estimation by GRE‐CEST‐1TE corrected the frequency drift, but less efficiently than the other two dynamic methods. Further investigations would be necessary to identify the source of this deviation; however, the known nonlinear phase evolution in white matter (due to specific tissue microstructure) could be a potential contributor.^51^

In our study, the accuracy of multiecho ∆*B*_0_ mapping was dependent not only on the ∆TE between the two echoes, as reported previously,^16^ but also on the actual values set for each TE and their receiver bandwidths. We had to optimize the TE settings experimentally to eliminate erroneous *B*_0_ offsets and spatially linear *B*_0_ gradients (mostly in the readout encoding direction) in phantoms prior to the CEST experiments. For routine use, it will be important to achieve accurate ∆*B*_0_ mapping for any CEST sequence setting without previous optimization. For similar reasons we also used only monopolar readout gradients, although GRE‐CEST‐2TE and GRE‐2TE should benefit from bipolar readout gradients. We have also refrained from performing additional corrections for any other confounding effects such as motion, *B*_1_ inhomogeneities, semisolid MT, water relaxation, T_2_‐dependent spillover or nuclear Overhauser enhancement exchange to prevent introducing factors that could make it difficult to isolate *B*_0_‐related effects.^52^, ^53^, ^54^, ^55^, ^56^, ^57^, ^58^, ^59^, ^60^ Of course these should be used when applying accurate CEST quantification in (patient) studies.

In the future, the proposed dynamic methods could be additionally combined with real‐time motion correction by extending the navigator to 3D as previously applied in MRI and MRSI.^47^, ^52^, ^53^, ^54^, ^55^ Thereby, artifacts due to motion and *B*_0_‐instabilities could be simultaneously mitigated. Although not investigated here, other CEST quantification routines such as Lorentzian or Bloch fitting^61^, ^62^, ^63^ would also be likely to benefit from the presented dynamic ∆*B*_0_ correction, since *B*_0_ is usually included as a fitting parameter.

## 6 CONCLUSION

We have presented three dynamic ∆*B*_0_ correction methods for CEST MRI that successfully mapped and compensated for *B*_0 _changes for each individual Z‐spectral point. Improved correction performance in the presence of frequency drift was demonstrated by comparison with established static approaches. Among them, the self‐correcting properties of CEST‐GRE‐2TE made it the most reliable and easiest to implement. Implementation of an interleaved navigator (NAV‐EPI‐2TE) was more complicated, but allowed improved dynamic ∆*B*_0_ correction even close to water, but not better than CEST‐GRE‐2TE for typically investigated frequency ranges.

Dynamic *B*_0_ corrections for CEST are another important step toward more reliable clinical CEST MRI without the need for (lengthy) prescans or for acquisition of additional Z‐spectral points near water (as required for CEST‐minZ), lending itself particularly to dynamic CEST MRI.
